# Investigation of Climate Change Impact on Water Resources for an Alpine Basin in Northern Italy: Implications for Evapotranspiration Modeling Complexity

**DOI:** 10.1371/journal.pone.0109053

**Published:** 2014-10-06

**Authors:** Giovanni Ravazzani, Matteo Ghilardi, Thomas Mendlik, Andreas Gobiet, Chiara Corbari, Marco Mancini

**Affiliations:** 1 Politecnico di Milano, Piazza Leonardo da Vinci, Milan, Italy; 2 Wegener Center for Climate and Global Change and Institute for Geophysics, Astrophysics, and Meteorology, University of Graz, Graz, Austria; University of Aveiro, Portugal

## Abstract

Assessing the future effects of climate change on water availability requires an understanding of how precipitation and evapotranspiration rates will respond to changes in atmospheric forcing. Use of simplified hydrological models is required beacause of lack of meteorological forcings with the high space and time resolutions required to model hydrological processes in mountains river basins, and the necessity of reducing the computational costs. The main objective of this study was to quantify the differences between a simplified hydrological model, which uses only precipitation and temperature to compute the hydrological balance when simulating the impact of climate change, and an enhanced version of the model, which solves the energy balance to compute the actual evapotranspiration. For the meteorological forcing of future scenario, at-site bias-corrected time series based on two regional climate models were used. A quantile-based error-correction approach was used to downscale the regional climate model simulations to a point scale and to reduce its error characteristics. The study shows that a simple temperature-based approach for computing the evapotranspiration is sufficiently accurate for performing hydrological impact investigations of climate change for the Alpine river basin which was studied.

## Introduction

According to the Fifth Assessment Report (AR5) of the United Nations Intergovernmental Panel on Climate Change (IPCC) [1], for average annual Northern Hemisphere temperatures, the period 1983–2012 was very likely the warmest 30-year period of the last 800 years. Climate change has significant implications for the environment [2], [3], water resources [4], and human life in general [5], which have motivated a multitude of scientific investigations over the past two decades [6], [7], [8], [9]. One of the expected impacts of climate change is a modification of water availability, due to the strict interaction between the climate system and the hydrological cycle. Therefore, an accurate assessment of the future effects of climate change requires an understanding of how precipitation and evapotranspiration rates will respond to changes in atmospheric forcing. The most common approach used to assess the hydrologic impact of global climate change involves climate models as input of hydrological models. In particular, the climate models simulate the climatic effects of increasing atmospheric concentrations of greenhouse gases, while the hydrological models are used to simulate the hydrological impacts of climate change [10]. River discharges, and their temporal distributions, are strongly affected by high mountainous areas [11], [12], which are particularly sensitive to global warming [13], [14]. The quality of hydrological impact investigations, even of larger catchments, thus depends on the capability to model those specific processes in mountainous regions.

The extreme complexity of the processes involved in the hydrology of mountainous areas, and the great spatial variability of meteorological forcings and river basin characteristics, require the use of physically based and spatially distributed hydrological models to simulate the transformation of rainfall into runoff [15], [16], [17]. Recent advances have made physically based hydrologic models more complex through the inclusion of more sophisticated land surface models, which compute the water and energy balances between the land surface and the atmosphere [18], [19]. This should improve the predictive skill, and facilitate the estimation of parameter values based on physiological characteristics or measurements. Conversely, more complex models suffer from computational requirements, which can limit their applicability when simulating long time series such as those required for climate change impact analyses. Moreover, in addition to precipitation and temperature data, more sophisticated models require, as an input, a complete dataset of meteorological forcings, including solar radiation, wind speed, and relative humidity. These variables may not be available, at proper spatial and temporal resolutions, to accurately capture the dynamics of the hydrological processes in mountainous areas [20]. As a consequence, the hydrological model used for the analysis of climate change impacts should be a compromise between its accuracy and its simulation time. This requires an assessment of the reliability of simplified hydrological models in contrast to the more sophisticated land surface models.

The main objective of this study was to quantify the differences between a simplified hydrological model, which computes the hydrological balance based on precipitation and temperature only, and an enhanced version of the model, which solves the energy balance to compute the actual evapotranspiration. The study was performed in three steps: first, the hydrological models were calibrated and validated against the river discharge measured in the control period; second, the hydrological models driven by climatic forcings were evaluated for their performance in reproducing the water balance components during the control period; and third, climate change impacts was assessed computing the differences between the hydrological variables simulated for the decade spanning 2041–2050 and those of the control period.

The structure of the paper is as follows. In section 2, description of the study area, and data and mathematical models used are presented. In section 3.1, the hydrological models driven by meterological forcings, are evaluated in reproducing the daily streamflow; in section 3.2 the hydrological models, driven by modelled climatic forcings, are evaluated in reproducing the hydrological aspects of the control period; in section 3.3 the climate change impacts on hydrological processes are presented. In the last section, conclusions are drawn.

## Data and Methods

### Study area

**Figure 1:**
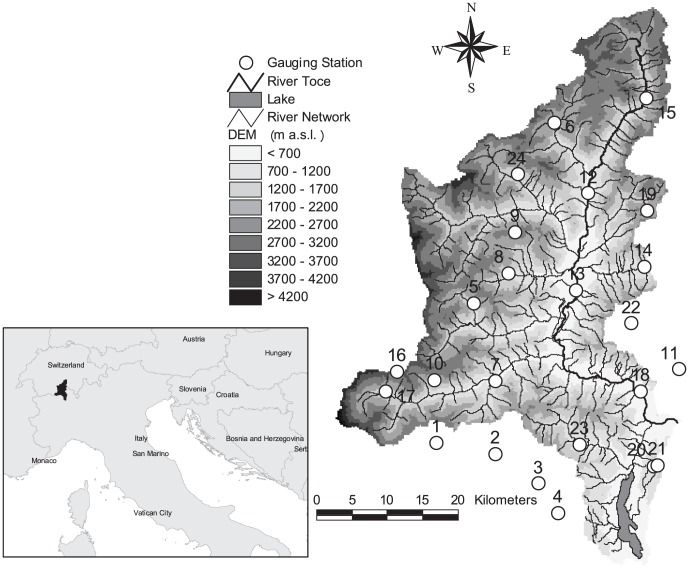
Localization of the stations on a DEM of the Toce watershed.

The Toce watershed is a typical glacial basin, with steep hillslopes bounding a narrow valley located primarily in the north Piedmont region of Italy, and partially in Switzerland (10% of the total area), and with a total drainage area of approximately 1,800 km^2^ (Fig. 1). Its elevation ranges from 193 m above sea level (a.s.l.) at the outlet to approximately 4,600 m a.s.l. at the Monte Rosa crest. The average elevation is 1,641 m a.s.l. Geographic coordinates of basin outlet are: 8.49027° longitude, 45.94028° latitude.

The land cover is composed of forests (70%), bare rocks (9%), agricultural land (7%), natural grassland (6%), urban centers (4%), bodies of water (3%), and glaciers and perpetual snow (1%). The Toce lithology has five main classes: augean gneiss (49%), micaceous schists (27%), calcareous schists (11%), grindstones (7%), and granites (6%). The steep hillslopes, forming the most significant area of the basin, are mostly covered by trees on thin soil layers resting on bedrock. The soil depth increases in the downstream narrow alluvial region where an unconfined aquifer interacts with the river course. Fourteen major dams are located within the Toce watershed, with a total effective storage capacity of approximately 151×10^6^ m^3^ [21].

A digital elevation model was available at a 200×200 meter resolution as retrieved from 1∶10,000-scale topographic maps [22]. The digital land use map was derived by coupling the CORINE (Coordination of Information on the Environment) land cover map [23] for the Italian portion, with the Swiss land use map (Arealstatistik) for the small portion of the basin located in Switzerland. Both the maps were derived from remote sensing observations [21]. Most of the parameter maps were produced during the European Union research project RAPHAEL (Runoff and Atmospheric Processes for flood HAzard forEcasting and controL), whose objective was to improve flood forecasting in the complex mountain watershed [24], [25].

The meteorological and hydrologic data were collected hourly by a telemetric monitoring system of the Regione Piemonte flood warning system. The data were available from January 1, 2000 to December 31, 2010 at the stations shown in Table 1 and Fig. 1 for the rainfall, air temperature, short wave solar radiation, air humidity, wind speed, and river discharges at Candoglia (1,534 km^2^ basin area). The mean value of the maximum annual flood peak is 944 m^3^/s, and the average discharge is 64 m^3^/s.

**Table 1:** Availability of data at the stations used in the hydrological analysis.

*ID*	*Name*	*Precipitation (mm)*	*Temperature (°C)*	*Radiation (w/m^2^)*	*Wind Speed (m/s)*	*Relative Humidity (%)*	*Discharge (m^3^/s)*
1	Carcoforo		X				
2	Fobello	X	X				
3	Sabbia	X	X				
4	Varallo	X	X			X	
5	Alpe Cheggio		X				
6	Alpe Devero	X	X			X	
7	Anzino	X	X				
8	Pizzanco	X	X			X	
9	Lago Paione	X	X	X		X	
10	Ceppo Morelli		X				
11	Cicogna	X	X				
12	Crodo	X	X			X	
13	Domodossola	X	X	X	X	X	
14	Druogno	X	X			X	
15	Formazza	X	X			X	
16	Passo Moro	X	X			X	
17	Pecetto	X	X				
18	Candoglia	X	X				X
19	Larecchio	X	X			X	
20	Baita CAI	X	X				
21	Mottarone	X	X			X	
22	Mottac	X	X				
23	Sambughetto	X	X				
24	Varzo	X	X			X	

### Two hydrological models with increasing complexity

Two distributed hydrological models were used for simulating the water balance components of the Toce river basin: the FEST-WB (Flash–flood Event–based Spatially distributed rainfall–runoff Transformation, including Water Balance [15], [26]) and the FEST-EWB (Flash–flood Event–based Spatially distributed rainfall–runoff Transformation, including Energy and Water Balance [27], [18]). The main difference between them is in the computation of evapotranspiration. The FEST-WB model derives the actual evapotranspiration by rescaling the potential evapotranspiration using a simple empirical approximation, where the potential evapotranspiration is computed based only on air temperature measurements. By contrast, the FEST-EWB model computes the actual evapotranspiration by solving the system of water mass and energy balance equations. The differences in the input parameters and meteorological forcings are listed in Table 2.

**Table 2:** Meteorological forcings and parameters used as input to the FEST-WB and FEST-EWB models.

*Input*	*Unit*	*FEST-WB*	*FEST-EWB*
Precipitation	mm	X	X
Temperature	°C	X	X
Solar Radiation	W/m^2^		X
Wind Speed	m/s		X
Relative Humidity	%		X
Saturated Hydraulic Conductivity	m/s	X	X
Residual Moisture Content	-	X	X
Saturated Moisture Content	-	X	X
Wilting Point	-	X	X
Field Capacity	-	X	X
Pore Size Index	-	X	X
Curve Number	-	X	X
Soil Depth	m	X	X
Vegetation Fraction	%	X	X
Crop Coefficient	-	X	
Leaf Area Index	m^2^/m^2^		X
Albedo	-		X
Minimum Stomatal Resistance	s/m		X
Vegetation Height	m		X

**Figure 2:**
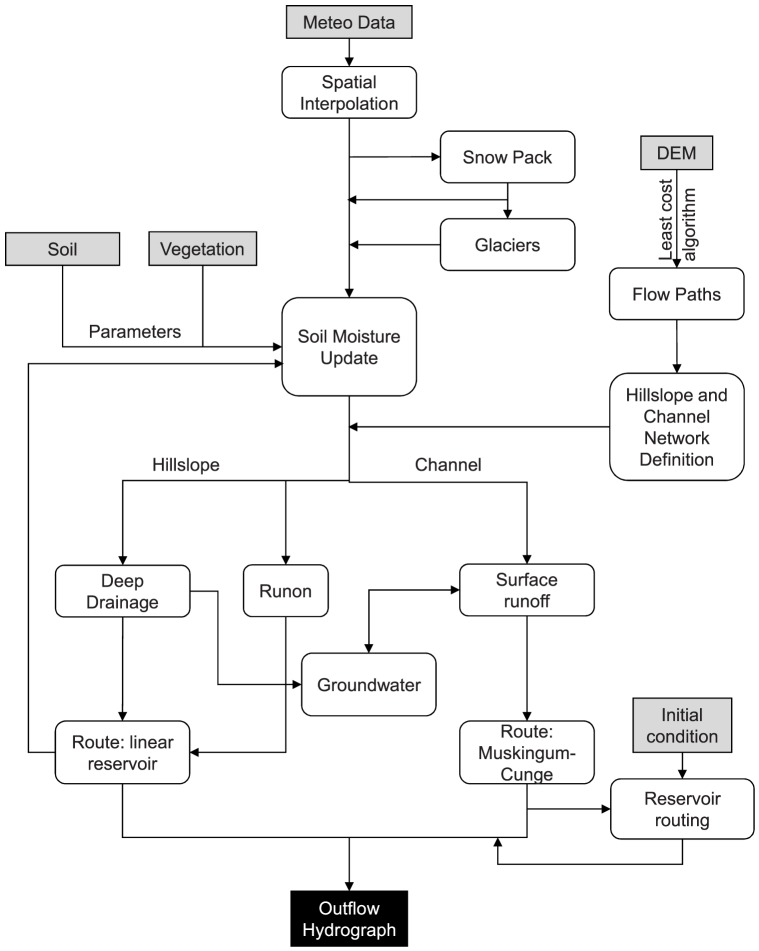
Scheme of the primary features common to the FEST-WB and FEST-EWB distributed-hydrological models.

Six principal components can be identified in all models (Fig. 2): 1) the flow paths and channel network definition; 2) the spatial interpolation of meteorological forcings; 3) the simulation of snow pack and glacier dynamics; 4) the estimation of losses and soil moisture updating; 5) the runoff and base flow routings, including the effect of artificial reservoirs; and 6) the groundwater and hyporheic exchanges with streamflow.

For further details on distributed hydrological models and their applications, the reader can refer to [28], [29], [30], [31], [32], [33], [34].

#### Actual evapotranspiration in the FEST-WB hydrological model

The global actual evapotranspiration rate is given by:
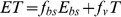
(1)where *E_bs_* is the actual rate of bare soil evaporation, *T* is the actual rate of transpiration, and *f_bs_* and *f_v_* are the fraction of the bare soil and the vegetation area, respectively (*f_bs_* + *f_v_* = 1). The actual rates of the bare soil evaporation and transpiration are computed as a fraction of the potential evapotranspiration, *PET*:
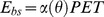
(2a)

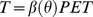
(2b)where
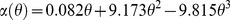
(3a)

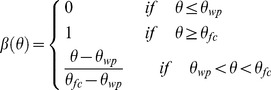
(3b)


and where 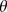 (-),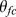 (-), and 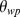 (-) are current water content, field capacity, and wilting point, respectively.

The potential evapotranspiration is given by
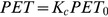
(4)where *K_c_* is the crop coefficient [35] retrieved from satellite images [36], [37], and *PET_0_* is the reference potential evapotranspiration that is computed with a temperature-based equation specifically developed for the Alpine environment [38]:
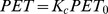


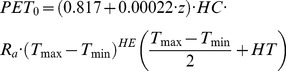
(5)


where *z* is the elevation (m a.s.l.), *R_a_* is the extraterrestrial radiation (mm·day^−1^), *T_max_* is the daily maximum air temperature (°C), *T_min_* is the daily minimum air temperature (°C), *HC* is the empirical coefficient (*HC* = 0.0023), *HE* is the empirical exponent (*HE* = 0.5), and *HT* is to convert units of Fahrenheit to Celsius (*HT* = 32/1.8 = 17.8) [39].

#### Actual evapotranspiration in FEST-EWB hydrological model

In the FEST-EWB model, the actual evapotranspiration is computed by solving the energy balance equation at the ground surface expressed as
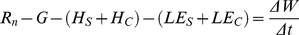
(6)where *R_n_* (W·m^−2^) is the net radiation, *G* (W·m^−2^) is the soil heat flux, *H_s_* and *H_c_* (W·m^−2^) and *LE_s_* and *LE_c_* (W·m^−2^) are the sensible heat and latent heat fluxes for the bare soil (*s*) and canopy (*c*), respectively, and Δ*W*/Δ*t* (W·m^−2^) assembles the energy storage terms. These terms are often negligible, especially with a low spatial resolution at the basin scale; however, the contribution of these terms can be significant at the local scale [40], [41]. *LE_c_* is a function of the canopy resistance, which is expressed as a function of the leaf area index, while *LE_s_* is a function of the soil resistance [18]. In this study leaf area index was retrieved from satellite images.

All of the terms of the energy balance depend on the land surface temperature (LST), which allows the energy balance equation to be solved by finding the thermodynamic equilibrium temperature which closes the equation using the Newton-Raphson method:
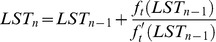
(7)where *LST_n_* is the actual value, *LST_n-_*_1_ is the value at the previous iteration, *f_t_*(*LST_n-_*_1_) is the energy balance function, and *f_t_*'(*LST_n-_*_1_) is its derivative. The solution is acceptable when 
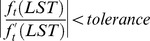
 and 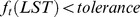, with *tolerance* equal to 0.001.

The FEST-EWB model has been proven to make accurate projections of the actual evapotranspiration against the energy and mass exchange measurements acquired by an eddy covariance station [18] and at the agricultural district scale against ground and remote sensing information [27].

### Calibration and validation of the hydrological models

The calibration of the snow module parameters was performed in a previous study described by [42] and [43]. Given that the first assigned values (based upon measured values or reference literature or an educated guess) provided satisfactory results in terms of time series discharge simulation, no other parameters were calibrated. The performance of the model was assessed by comparing the daily simulated and observed discharge at Candoglia in the period from 2001 to 2010. The year 2000 was treated as the period for the model initialization. The performance of the models was assessed through two goodness of fit indices, the Root Mean Square Error (RMSE) and the Nash and Sutcliffe [44] efficiency (*η*), defined as follows:
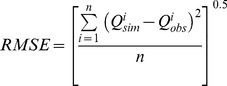
(8)




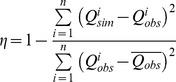
(9)


where *n* is the total number of time steps, 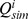 is the *i*th simulated discharge, 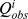 is the *i*th observed discharge, and 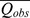 is the mean of the observed discharges.

### At-site bias-corrected climate-scenario forcings

For the meteorological forcing of future scenarios, two different regional climate models (RCMs) were used, the REMO [45] and the RegCM3 [46]. Both models cover Europe on a 25×25 km grid, in the same simulation period (1951–2100). Moreover, they are driven by the same global ocean-atmosphere-coupled model, ECHAM5 [47], using the observed greenhouse gas concentrations between 1951 and 2000 and IPCC's (Intergovernmental Panel on Climate Change) greenhouse gas emission scenario A1B [48] between 2001 and 2100. Both were produced within the EU FP6 Integrated Project ENSEMBLES (http://www.ensembles-eu.org/) and can be downloaded from http://ensemblesrt3.dmi.dk on a daily basis. Hourly and 3-hourly data were provided directly by the Max Planck Institute for Meteorology and the Abdus Salam International Center for Theoretical Physics. In comparison to the larger ensemble of regional simulations for Europe, the REMO and RegCM3 models represent moderate warming (below average) and near-average precipitation changes [49].

A quantile-based error-correction approach (quantile mapping) has been used to downscale the RCM simulations to a point scale and to reduce its error characteristics. The potential of quantile mapping for correcting GCM data has already been demonstrated in previous hydrological studies [50], [51], but its application to regional climate simulations is somewhat recent [52], [53], [54], [55], [56], [57]. In this study, the quantile mapping applied observational stations data to climate data, from the regional climate models, on a daily basis. It adapted the modelled time series to the observed empirical cumulative frequency distribution [58]. The method and its application were discussed by [59] and [54] as to what concerns daily temperature and precipitation, and by [60] as regards other meteorological variables such as relative humidity, global radiation, and wind speed. All the variables used in this study were error corrected and downscaled to a station basis. A 31-day moving window in the calibration period, centered on the day to be corrected, was used for constructing the empirical cumulative frequency distribution for that particular day of the year. This enabled an annual cycle-sensitive correction as well as a sufficiently large sample size. A point-wise implementation, which fits a separate statistical model for each observational station, was chosen to account for the regionally varying errors. Grid cell averages (3×3) of the raw RCM data were used as predictors with respect to the effective resolution of the RCM, which is below the grid-resolution.

The calibration period for the error correction ranged from 01-01-2000 to 12-31-2009. No error correction was performed for stations with less than 9 years of observational data (>10% missing data), because the climate variability could not be expected to be properly covered by only a few years of data. Quantile mapping assumes that the same statistical relations of the observed and modelled climate hold within the calibration period, as well as in future scenario periods. It must be kept in mind that even a calibration period of 9–10 years can be affected by decadal climate variability, which can degrade the results of the error correction applied to the future scenario period.

The resulting daily scenarios were further refined to a 3-hourly time series using the sub-daily data from the RCMs. For the air temperature, the differences between the 3-hourly RCM data and their daily-mean values were added to the corresponding corrected-daily values. The ratios of the 3-hourly RCM data and daily precipitation values were multiplied by the corrected daily values. Similarly, for the global radiation, the wind speed, and the relative humidity, the ratios of the 3-hourly RCM data and their daily mean values were multiplied by the corrected daily value. In the case of relative humidity values exceeding 100%, all of the values from the day were multiplied by a factor to shrink the daily maximum value to 100%.

Climate-scenario dataset used in this analysis is included as supplemental file (Dataset S1).

## Results and Discussion

### Comparison of the models driven by meteorological observations

**Figure 3:**
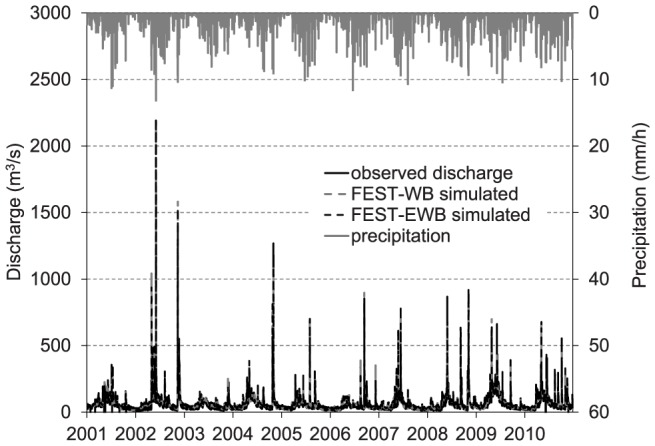
Comparison between the simulated and observed hourly discharge from the FEST-WB and FEST-EWB hydrological models.

Table 3 shows the results in reproducing the daily streamflow by the FEST-WB and FEST-EWB models forced by meteorological observations. The goodness of fit indices of the two models are comparable, with the FEST-WB model displaying a slightly greater Nash and Sutcliffe efficiency and lower RMSE. In Fig. 3, a comparison between the FEST-WB and FEST-EWB models for the simulated and observed hourly discharge is shown for the period from 2001 to 2010. The differences were almost negligible and the time series overlapped.

**Table 3:** Root mean square error (*RMSE*), m^3^/s, and Nash and Sutcliffe efficiency (*η*) for the FEST-WB and FEST-EWB driven by observed meteorological forcings.

*Index*	*FEST-WB*	*FEST-EWB*
*RMSE*	26.7	29.5
*η*	0.81	0.76

**Figure 4:**
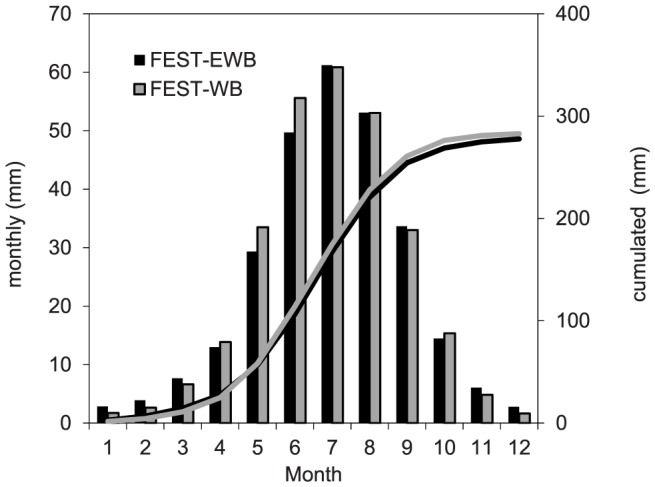
Mean monthly and cumulated actual evapotranspiration as computed by the FEST-WB and FEST-EWB hydrological models driven by meteorological observations.

The mean monthly and cumulated actual evapotranspiration values, as computed by the FEST-WB and FEST-EWB models driven by meteorological observations, are shown in Fig. 4. There is a general agreement between the two different approaches in computing the evapotranspiration with the exceptions of May and June, when the FEST-WB model had values 14% and 12% higher, respectively, than the FEST-EWB model. On an annual basis, the FEST-WB model had a 1.8% higher value than the FEST-EWB model.

**Figure 5:**
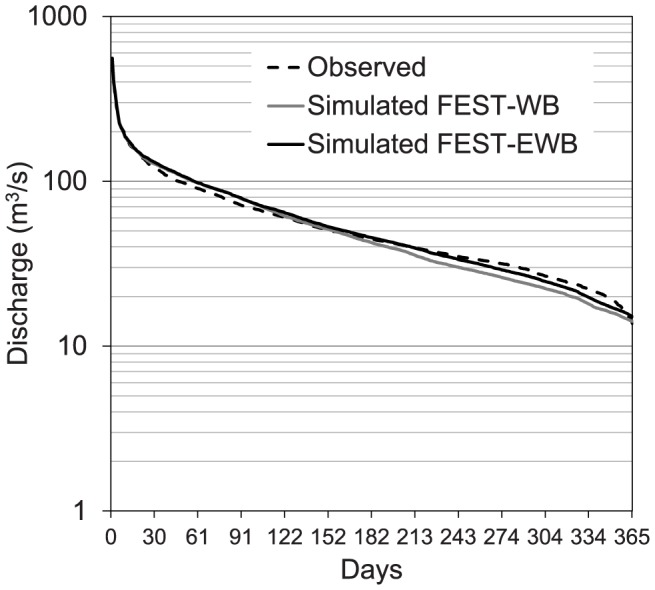
Mean flow duration curves for 2001–2010 from the observed discharges and those simulated by the FEST-WB and FEST-EWB hydrological models driven by meteorological observations.

In Fig. 5, the mean flow duration curves simulated by the two models are compared to those observed. Good agreement was seen for both the higher and lower discharges. The difference between the FEST-WB and FEST-EWB simulations was due to the different evapotranspiration losses reported by the two models.

### Comparison of models in reproducing the hydrological aspects of the control period

Before assessing the climate change impacts, we performed an analysis of the hydrological models, driven by modelled climatic forcings, in reproducing the hydrological aspects of the control period (2001–2010). Table 4 shows the mean annual precipitation and the average daily mean, maximum, and minimum temperatures observed and simulated by the calibrated REMO and RegCM3 climate models during the control period. The REMO model resulted in a 0.11°C and 0.95% underestimation in reproducing the temperature and precipitation, respectively, while the RegCM3 model resulted in a 0.08°C and 5.2% underestimation.

**Table 4:** Daily mean (*T*), maximum (*T_max_*) and minimum (*T_max_*) temperature and mean annual precipitation (*P*) observed and simulated by error corrected REMO and RegCM3 climate models for control period (2001–2010).

	*T (°C)*	*T_max_ (°C)*	*T_min_ (°C)*	*P (mm)*
observed	4.17	8.14	0.52	1412.75
REMO	4.06	8.33	0.46	1399.25
RegCM3	4.09	7.04	1.33	1339.04

The two climate models displayed larger differences in reproducing the daily maximum and minimum temperature. The REMO model produced errors of 0.2°C and −0.1°C in reproducing the maximum and minimum daily temperatures, respectively, while the RegCM3 model produced errors of −1.1°C and 0.8°C, respectively. This can be explained by the fact that only the daily mean values were error corrected, while the diurnal cycle was superimposed as the models simulated it without further correction (Section 2.4).

**Figure 6:**
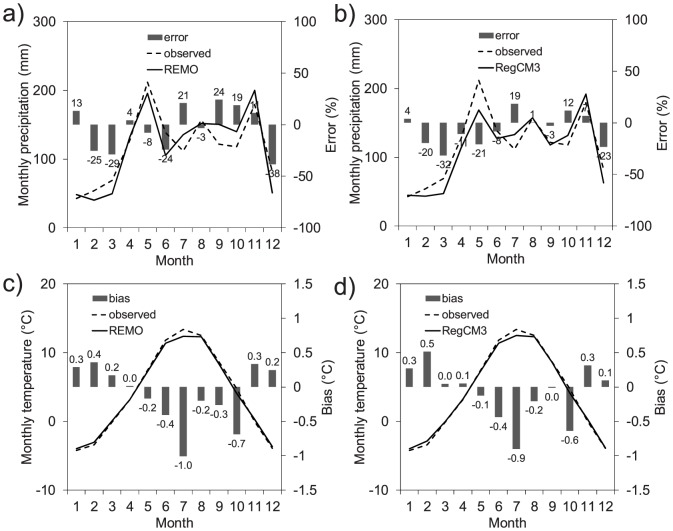
Mean monthly precipitation and temperature for 2001–2010 as simulated by the REMO and RegCM3 regional climate models and their deviations versus the observations.

In Fig. 6, the mean monthly precipitation and temperature, as simulated by the REMO and RegCM3 climate models, are compared to those observed. Regarding precipitation, the two models underestimated it in February, March, May, June, and December and overestimated it in July, October, and November. Nevertheless, the overall behavior, such as the peaks in the spring and autumn, was well captured. The two climatic models displayed the same results in reproducing the monthly temperatures, that is, they underestimated it from May to October and overestimated it in the other months of the year, but the discrepancies were under the acceptable limits.

Since the evaluation period differs from the calibration period of the bias correction, such minor discrepancies should be expected. An increase of these errors in future applications should also be expected. However, this increase can be regarded as limited, given that the bias correction is relatively stable, as has been demonstrated by [61] and [60].

**Figure 7:**
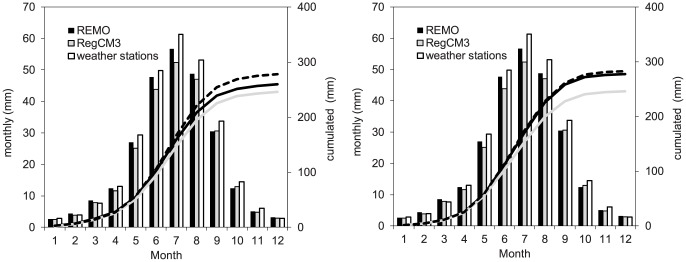
Mean monthly and cumulated actual evapotranspiration as computed for 2001–2010 by the FEST-EWB (left) and FEST-WB (right) hydrological models driven by the REMO and RegCM3 regional climate models and the weather observations during the control period (2001–2010).

Fig. 7 shows the mean monthly and cumulated actual evapotranspiration as computed by the FEST-EWB and FEST-WB hydrological models driven by REMO, RegCM3, and meteorological observations during the control period. Both the FEST-EWB and FEST-WB models simulated greater evapotranspiration when driven by REMO than by RegCM3. The FEST-WB equation to compute the evapotranspiration is very sensible to the daily temperature range. This implies that the evapotranspiration computed by the FEST-WB model, driven by REMO, matches the evapotranspiration computed by the FEST-WB model, driven by the meteorological observations, as the REMO model is more accurate than the RegCM3 model in reproducing the daily minimum and maximum temperatures.

**Figure 8:**
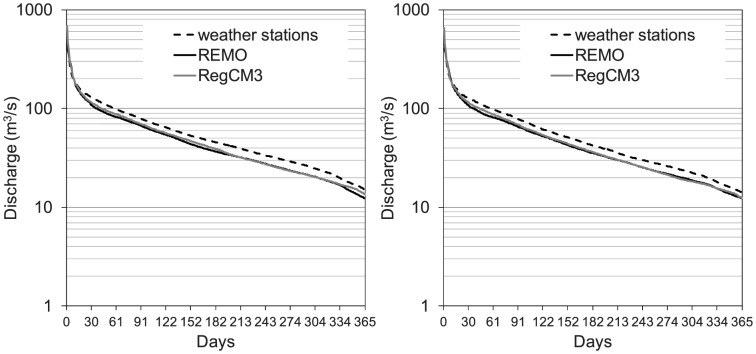
Mean flow duration curves simulated by the FEST-EWB (left) and FEST-WB (right) hydrological models driven by meteorological observations and the simulated climatic forcings by the REMO and RegCM3 regional climate models.

Fig. 8 shows the mean flow duration curves simulated by the FEST-EWB and FEST-WB hydrological models driven by meteorological observations and the REMO and RegCM3 simulated climatic forcings. There was good agreement between the REMO and RegCM3 driven simulations. The discharges driven by the RegCM3 model were generally greater than those driven by REMO, particularly for the high durations, due to the underestimation of the evapotranspiration component.

### Projected changes in the hydrological processes

The impacts of climate change on hydrological processes were assessed by comparing the results from the FEST-EWB and FEST-WB models, driven by the REMO and RegCM3 climate models, for the decade spanning 2041–2050 to those of the control period. The simulations were performed assuming no variation in the spatial distribution of the vegetation, or the beginning and duration of the growing season. This means that the crop coefficient functions for the future period were unmodified, and that the monthly leaf area index maps were derived as an average from the 2001–2010 remotely sensed maps.

Table 5 shows the average daily mean, maximum, and minimum temperatures and the mean annual precipitation simulated by the REMO and RegCM3 models for the 2041–2050 decade. The REMO and RegCM3 models simulated an increase in mean temperature of 1.28°C and 1.12°C, respectively, and an increase in the mean annual precipitation of 12.83% and 25.35%, respectively.

**Table 5:** Daily mean (*T*), maximum (*T_max_*) and minimum (*T_max_*) temperature and mean annual precipitation (*P*) simulated by REMO and RegCM3 climate models for decade 2041–2050.

	*T (°C)*	*T_max_ (°C)*	*T_min_ (°C)*	*P (mm)*
REMO	5.35	9.01	1.34	1678.54
RegCM3	5.21	7.88	2.16	1578.85

**Figure 9:**
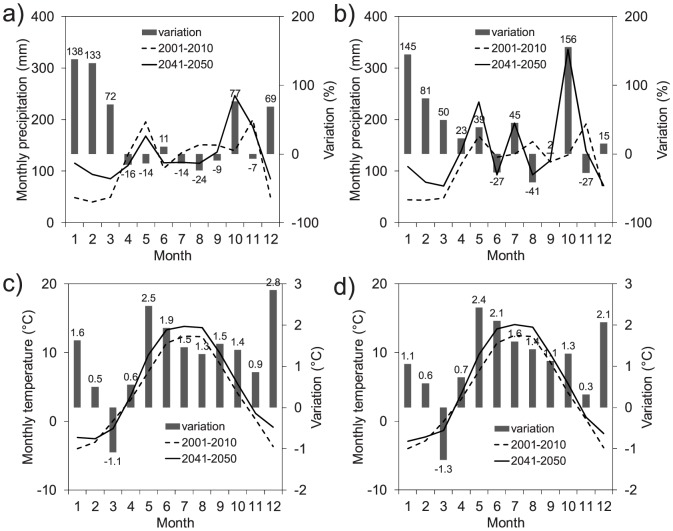
Mean monthly precipitation and temperature for the period 2041–2050 as projected by REMO and RegCM3 regional climate models versus the control period (2001–2010): (a) precipitation by REMO; (b) precipitation by RegCM3; (c) temperature by REMO; and (d) temperature by RegCM3.

The mean monthly precipitation and temperature as simulated by REMO and RegCM3 for the control period (2001–2010) and for the 2041–2050 decade are shown in Fig. 9. The precipitation increase was mostly concentrated in the winter period and October, in which the RegCM3 model projected a precipitation increase of 156%. During the summer, the two climate models projected a significant decrease in precipitation, as much as −41% in August by the RegCM3 model. The temperature was generally predicted to increase more significantly during the summer, late spring, and winter, while a decrease was expected in March. These results are consistent with findings depicted in the IPCC AR4 for Central Europe [62] and with a recent review on expected climate change in the Alpine region [14].

**Figure 10:**
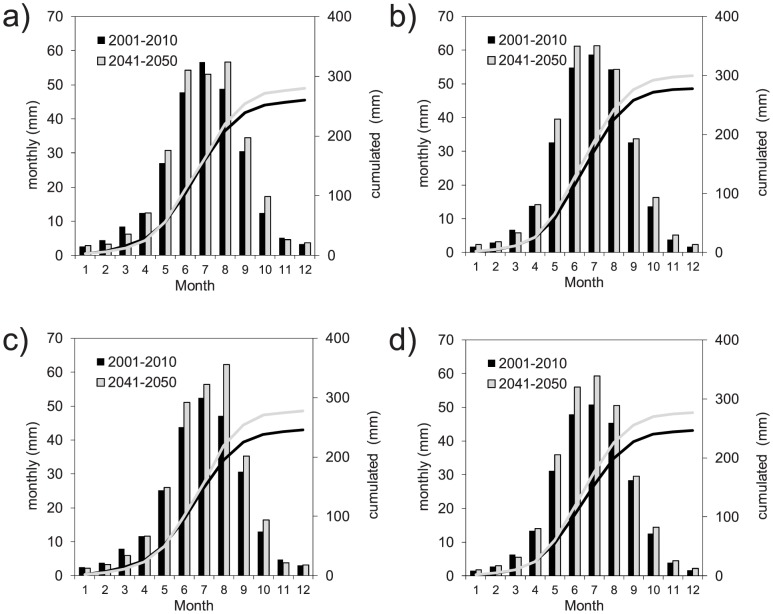
Mean monthly and cumulated actual evapotranspiration for the period 2041–2050 as simulated by the FEST-WB and FEST-EWB hydrological models driven by the REMO or RegCM3 regional climate models versus the control period (2001–2010): (a) FEST-EWB driven by REMO; (b) FEST-WB driven by REMO; (c) FEST-EWB driven by RegCM3; and (d) FEST WB driven by RegCM3.

Fig. 10 shows the mean monthly and cumulated actual evapotranspiration as computed by the FEST-EWB and FEST-WB models driven by REMO and RegCM3 for the control period (2001–2010) and the 2041–2050 decade. All of the simulations showed an increase in the evapotranspiration, in agreement with the increase in air temperature. For a given climate model, the FEST-EWB and FEST-WB hydrological models projected similar modifications to the evapotranspiration.

**Figure 11:**
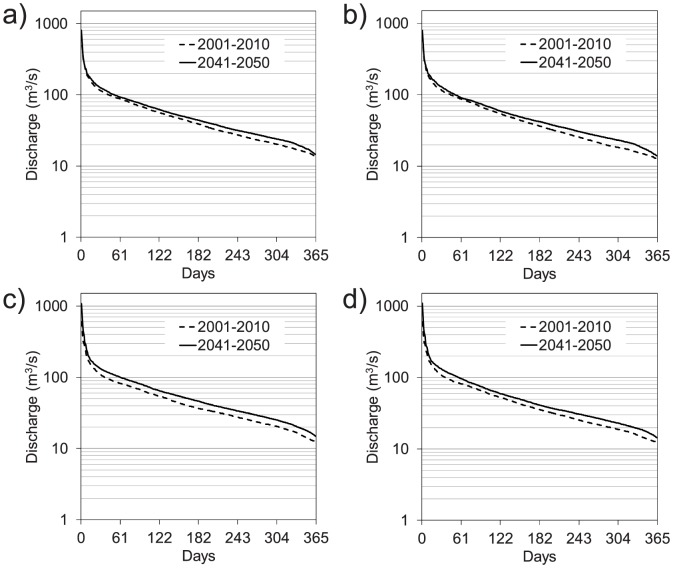
Mean flow duration curve for the period 2041–2050 as simulated by the FEST-WB and FEST-EWB hydrological models driven by the REMO or RegCM3 regional climate models versus the control period (2001–2010): a) FEST-EWB driven by REMO, b) FEST-WB driven by REMO, c) FEST-EWB driven by RegCM3, and d) FEST WB driven by RegCM3.

In Fig. 11, the mean flow duration curves simulated by the FEST-EWB and FEST-WB hydrological models, driven by REMO and RegCM3 for the 2041–2050 decade, were compared to those of the control period (2001–2010). A general increase of the discharge was projected for the flow duration, in agreement with the significantly increased annual precipitation not compensated by increased evapotranspiration. For a given climate model the FEST-EWB and FEST-WB hydrological models predicted similar modifications to the flow duration curve.

**Figure 12:**
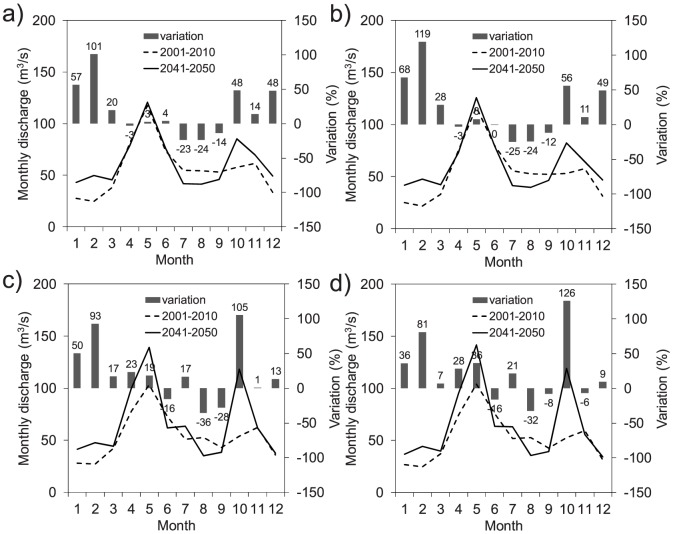
Mean monthly discharge for the period 2041–2050 as simulated by the FEST-WB and FEST-EWB hydrological models driven by the REMO or RegCM3 regional climate models versus the control period (2001–2010): a) FEST-EWB driven by REMO, b) FEST-WB driven by REMO, c) FEST-EWB driven by RegCM3, and d) FEST WB driven by RegCM3.

In Fig. 12, the mean monthly discharge for the 2041–2050 decade, simulated by the FEST-EWB and FEST-WB hydrological models driven by the REMO and RegCM3 climate models, is compared to that of the control period (2001–2010). The seasonal shift observed in the precipitation was reflected in the projected monthly discharge, with a significant increase expected in October and in the winter period and a significant decrease expected in summer.

## Conclusions

This study investigated the role of climatic forcing availability, and thus hydrological model complexity, on the assessment of climate change impacts on the water resources for the Toce river basin. Two distributed hydrological models were used to simulate the water balance components of the Toce river basin: the FEST-WB model, which implements a simple temperature-based method for computing the evapotranspiration; and the FEST-EWB model, which computes evapotranspiration by solving energy and water balance equations that require temperature, net radiation, wind speed, and relative air humidity as meteorological forcings. Both the FEST-WB and FEST-EWB models performed well in reproducing the daily discharge of the 2001–2010 period and the hourly discharge for major flood events. The difference in computing the evapotranspiration was approximately 2% on an annual basis. Moreover, there was general agreement between the two hydrological models in reproducing the mean annual flow-duration curve.

An analysis of the hydrological models, driven by the climatic forcings modelled by the REMO and RegCM3 climate models, in reproducing the hydrological aspects of the control period showed that the FEST-WB model was more sensitive to the daily temperature range in simulating the evapotranspiration. The evapotranspiration differences impacted the flow duration curve, but the two hydrological models achieved good agreement.

The impact of climate change on the hydrological processes was assessed by comparing the results from the FEST-EWB and FEST-WB models, driven by REMO and RegCM3 for the 2041–2050 decade, to those of the control period (2001–2010). The REMO and RegCM3 climate models simulated increased mean temperatures, and increased mean annual precipitations. The precipitation increase was primarily concentrated during October and the winter period. The two climate models predicted a significant decrease in precipitation during the summer. This reflects an increase in the evapotranspiration and the discharges for all of the durations in the flow duration curves. The seasonal shift observed in the precipitation was reflected in the monthly discharge. Indeed, a significant increase in the discharge was expected in October and the winter period, while a significant decrease was expected in the summer. Obtained results are generally consistent with findings depicted in the IPCC assessment reports.

In general, this study showed that despite the simple temperature-based approach for computing evapotranspiration, the FEST-WB model is robust and sufficiently accurate to perform hydrological impact studies of climate change for the Alpine river basin that was investigated. The bias introduced by the approximations from the method used to compute the evapotranspiration was less than the uncertainty associated with climate models.

## Supporting Information

Dataset S1 **At-site bias-corrected climate data used in the hydrological models for the period 1951–2050, which include 3-hourly data for rainfall in mm (PRE_3hc.txt), air temperature in °C (TAS_3hi.txt), wind speed in m/s (WSS_3hi.txt), solar radiation in W/m^2^ (RSDS_3hi.txt), and percentage relative humidity (HURS_3hi.txt).**
(ZIP)
